# Tumor Necrosis Factor-α Receptor 1 Mediates Borna Disease Virus 1-Induced Changes in Peroxisomal and Mitochondrial Dynamics in Neurons

**DOI:** 10.3390/ijms25031849

**Published:** 2024-02-03

**Authors:** Dominic Osei, Eveline Baumgart-Vogt, Barbara Ahlemeyer, Christiane Herden

**Affiliations:** 1 Institute for Anatomy and Cell Biology, Justus Liebig University Giessen, 35392 Giessen, Germany; dominic.osei@vetmed.uni-giessen.de (D.O.); eveline.baumgart-vogt@anatomie.med.uni-giessen.de (E.B.-V.); 2 Institute of Veterinary Pathology, Justus Liebig University Giessen, 35392 Giessen, Germany; 3 Center for Mind, Brain and Behavior, Justus Liebig University Giessen, 35392 Giessen, Germany

**Keywords:** Borna disease virus 1, persistent infection, brain, mitochondria, peroxisomes, catalase, SOD2, TNF, TNFR1, TNFR2

## Abstract

Borna disease virus 1 (BoDV1) causes a persistent infection in the mammalian brain. Peroxisomes and mitochondria play essential roles in the cellular antiviral immune response, but the effect of BoDV1 infection on peroxisomal and mitochondrial dynamics and their respective antioxidant capacities is still not clear. Using different mouse lines—i.e., tumor necrosis factor-α transgenic (TNFTg; to pro-inflammatory status), TNF receptor-1 knockout (TNFR1ko), and TNFR2ko mice in comparison to wild-type (Wt) mice—we analyzed the abundances of both organelles and their main antioxidant enzymes, catalase and superoxide dismutase 2 (SOD2), in neurons of the hippocampal, cerebral, and cerebellar cortices. In TNFTg mice, a strong increase in mitochondrial (6.9-fold) and SOD2 (12.1-fold) abundances was detected; meanwhile, peroxisomal abundance increased slightly (1.5-fold), but that of catalase decreased (2.9-fold). After BoDV1 infection, a strong decrease in mitochondrial (2.1–6.5-fold), SOD2 (2.7–9.1-fold), and catalase (2.7–10.3-fold) abundances, but a slight increase in peroxisomes (1.3–1.6-fold), were detected in Wt and TNFR2ko mice, whereas no changes occurred in TNFR1ko mice. Our data suggest that the TNF system plays a crucial role in the biogenesis of both subcellular organelles. Moreover, TNFR1 signaling mediated the changes in peroxisomal and mitochondrial dynamics after BoDV1 infection, highlighting new mechanisms by which BoDV1 may achieve immune evasion and viral persistence.

## 1. Introduction

BoDV1 is an enveloped, non-segmented, single negative-stranded RNA virus belonging to the family Bornaviridae of the order Mononegavirales; this virus replicates in the nucleus of target cells and causes a severe neurological disorder named Borna disease (BD) [1,2,3,4,5]. After nasal exposure of susceptible end hosts, BoDV1 primarily spreads to the limbic system, and eventually to the entire central nervous system (CNS), resulting in the release of pro-inflammatory cytokines like TNF, interleukin (IL)-1, and IL-2, followed by severe non-purulent meningoencephalitis [4,5,6,7,8]. BoDV1 infects a wide range of warm-blooded animals including humans, though severe disease occurs mainly in sheep and horses with a mortality rate of approximately 90% [9,10,11,12]. Experimental BoDV1 infection of neonatal mice causes neurological signs by 28–42 days post-infection (dpi), characterized by weight loss, ataxia, paraparesis, torticollis, nervous ticks, coarse fur, and death associated with a non-purulent meningoencephalitis in susceptible mouse strains [3,8,13]. Susceptibility to BoDV1 was in the case of wild-type mice was highest for Murphy Roth large (MRL) mice, medium for Balb/c mice, and lowest for C57/Bl6 mice, where 83%, 37%, and 13% of infected animals developed neurological symptoms, respectively. In the less susceptible C57/Bl6 mice, this percentage increased up to 100% and 30% after changes in the immune system were induced via IL-12 [14] or TNF overexpression [8], respectively. In our study, a mouse-adapted virus strain was injected into the brains of C57/Bl6 mice, which is reliably able to induce disease in TNFTg mice, as shown before [8,15]. In neonatal rats, abnormalities in learning and behavior are observed despite a lack of inflammation in the brains of asymptomatic animals [5,7,16]. In humans, BoDV1 infections—most likely transmitted by the bicolored, white-toothed shrew (reservoir host)—lead to fatal encephalitis; so far, about 45 cases have been diagnosed in Germany [12,17,18,19]. Presumably, the number of unreported cases is higher as testing for BoDV1 infection is not included in clinical screening protocols. Given the mounting reports on drastic BD cases in humans [9,10,11] and the lack of curative treatment options, there is the need for greater insights into the pathogenesis of BoDV1-induced encephalitis. Only a few reports have shown putative mechanisms for how BoDV1 sustains infection in the mammalian brain, acting on different molecules and pathways [12]. For instance, the P protein of BoDV1 binds TANK-binding kinase 1 and thereby suppresses type I interferon (IFN) production, stifling innate antiviral signaling [20]. In addition, the BoDV1 X protein was demonstrated to inhibit rotenone-induced axonal fragmentation, protecting neurons from degeneration and enhancing viral replication and spread [21].

TNF is a pleiotropic molecule of the immune system and belongs to the TNF/TNFR superfamily [8,22,23]. TNFR1, found on almost all cell types of the CNS, is primarily stimulated by soluble TNF, leading to pro-inflammatory and apoptotic signaling, whereas TNFR2, found on immune cells, endothelial cells, and a subset of neurons, is activated by transmembrane TNF, evoking anti-inflammatory and pro-survival effects [23,24]. In the normal CNS, TNF expression is low and localized mainly to microglia, astrocytes, and neurons, but is markedly elevated during and after injuries (e.g., ischemia, trauma, and infection) due to secretion mainly by microglial cells [22]. Soluble TNF binds to TNFR1, leading to pro-inflammatory and apoptotic signaling, whereas TNFR2, found on immune cells, endothelial cells, and a subset of neurons, is activated by transmembrane TNF, evoking anti-inflammatory and pro-survival effects [23,24]. In vivo, the prolonged exposure of brain cells to high levels of TNF mimics chronic CNS inflammation with white matter degeneration [25]. In the case of moderate TNF overexpression—as occurs in TNFTg mice—non-purulent meningoencephalitis typified by spontaneous epileptic seizures was seen after BoDV1 infection, whereas Wt mice did not show any inflammation [8,15].

In addition to the typical antiviral pathways and molecules of the innate immune system, virus-induced changes in subcellular organelles such as peroxisomes and mitochondria remain largely unexplored. Both organelles influence intracellular redox homeostasis by producing and detoxifying reactive oxygen species (ROS) [26,27,28]. Peroxisomes generate hydrogen peroxide (H_2_O_2_) during β-oxidation of very-long-chain fatty acids (VLCFAs); afterwards, H_2_O_2_ is detoxified by the peroxisomal matrix enzyme catalase into water and oxygen [29,30,31]. Peroxisomes harbor additional antioxidant enzymes such as SOD1, glutathione peroxidase, peroxiredoxin 5, and glutathione S-transferase [32,33]. Mitochondria generate ATP via the tricarboxylic cycle and oxidative phosphorylation, and are considered the major source of ROS, namely superoxide. Superoxides are detoxified by SOD2 into H_2_O_2_, which is further converted to oxygen and water by glutathione peroxidase 1 within mitochondria. Other antioxidant enzymes within mitochondria include thioredoxinreductase 2, thioredoxin 2, peroxiredoxins 3 and 5, and SOD1 [34].

Peroxisomes either synthesize pro-inflammatory (prostaglandins, leukotrienes, thromboxanes) or anti-inflammatory lipid derivatives (resolvins, maresins, protectins) [30]. In addition, some peroxisomal-derived molecules (ROS, free fatty acids, polyamines) stimulate immune cells, while others (oxysterols) suppress inflammation [30]. On the outer membranes of peroxisomes and mitochondria, adaptor proteins, known as mitochondrial antiviral signaling proteins (MAVSs), interact with retinoic-acid-inducible gene I (RIG-I)-like receptors (RLRs), to detect virus RNAs that enter host cells [26,28,35]. After binding of RLRs to MAVS, the latter becomes activated, leading to type I and III IFN responses, accompanied by the release of cytokines for recruiting immune cells and creating an antiviral state within the host cell [26,28,35]. The X protein of BoDV1 has been shown to bind to MAVS, inhibiting MAVS-mediated signaling and neuronal apoptosis to promote viral replication and persistence [36].

However, until now, there has been no comprehensive study analyzing changes in the numerical abundances of peroxisomes and mitochondria and their respective main antioxidant enzymes, catalase and SOD2, after BoDV1 infection. In this study, we used three different mouse models to investigate the role of TNF and its receptors, namely TNFTg (where the TNF gene is overexpressed at moderate levels under the control of the N-methyl-D-aspartate 2b receptor subtype (NR2B) promoter) [8,24,37,38], TNFR1ko, and TNFR2ko mice. We analyzed peroxisomal and mitochondrial abundances and their main antioxidant enzymes in the hippocampal, cerebral, and cerebellar cortices of non-infected and BoDV1-infected mice. Our study aimed to understand the effects of BoDV1 infection on the abundances of both subcellular compartments as well as the antioxidant enzymes, catalase and SOD2, whilst observing the role (s) of the TNF system in BoDV1 infection events of the brain. Our results might explain another piece in the puzzle concerning how BoDV1 is able to achieve and maintain persistence in the CNS, revealing TNFR1 signaling as a crucial mediator. These results provide a headway into future studies to develop therapeutic strategies against BD, e.g., by using drugs affecting organelle abundance or the antioxidant system in the CNS.

## 2. Results

### 2.1. Differences in Abundances of Peroxisomes, Mitochondria, Catalase, and SOD2 in Different Brain Regions of Non-Infected Wt Mice

**Figure 1:**
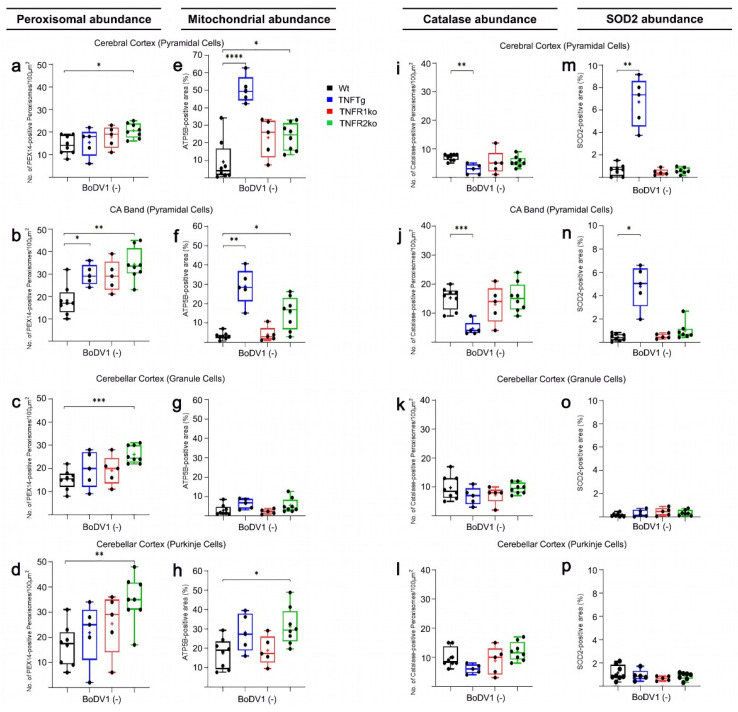
Differential changes (one-way ANOVA) in peroxisomal, mitochondrial, catalase, and SOD2 abundances. Immunofluorescent signals of PEX14 (peroxisomal marker; (**a**–**d**)), ATP5B (mitochondrial marker; (**e**–**h**)), catalase (**i**–**l**), and SOD2 (**m**–**p**) were quantified in distinct neuronal cell types in non-infected mice of each mouse line (Wt (black), TNFTg (blue), TNFR1ko (red) and TNFR2ko (green) mice). Wt, wild-type; TNFTg, TNF transgenic; TNFR1ko, TNF receptor 1 knockout; TNFR2ko, TNF receptor 2 knockout; BoDV1(−), without BoDV1 infection; CA, cornu ammonis band. Values are means ± SD; * *p* < 0.05, ** *p* < 0.01, *** *p* < 0.001, **** *p* < 0.0001 different from the respective control group.

**Figure 2:**
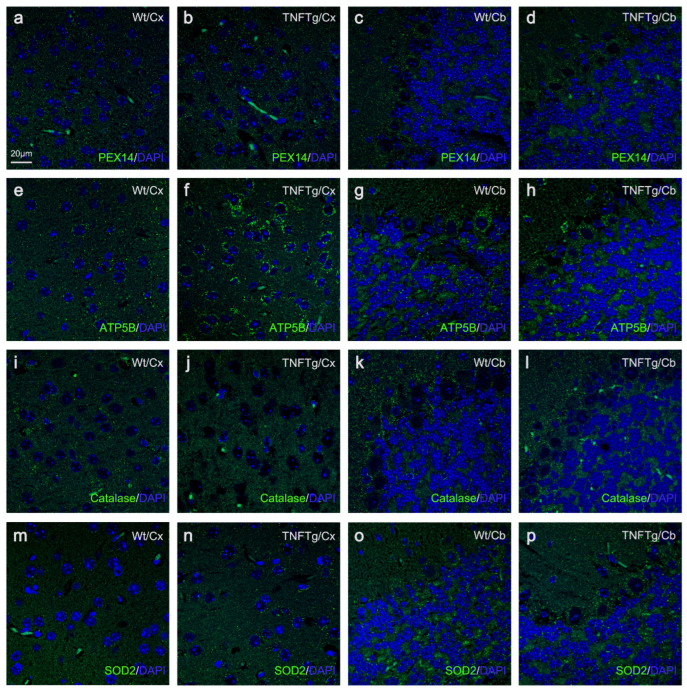
Immunofluorescence photomicrographs of PEX14- and catalase-positive peroxisomes (green) and ATP5B- and SOD2-positive mitochondria (green) in pyramidal neurons of the cerebral cortex and cerebellar granule and Purkinje neurons of non-infected Wt and TNFTg mice. Using fluorescing antibodies, 2 µm thick brain sections were indirectly immunostained against PEX14 (peroxisomal marker; (**a**–**d**)), ATP5B (mitochondrial marker; (**e**–**h**)), catalase (**i**–**l**), and SOD2 (**m**–**p**). DAPI (blue) was used as the nuclear stain for all neuronal cell types. Wt, wild-type; TNFTg; TNF transgenic; Cx, cerebral cortex; Cb, cerebellar cortex.

**Figure 3:**
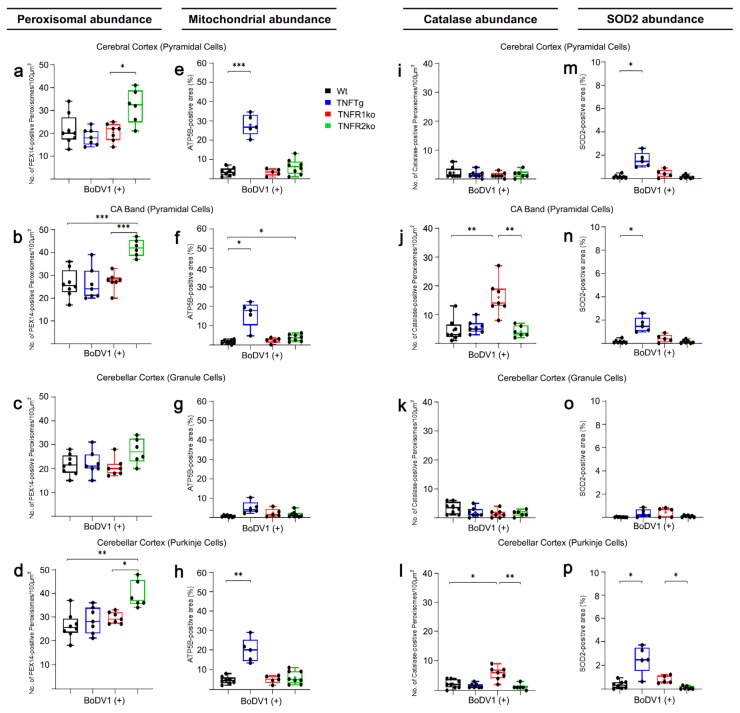
Differential changes (one-way ANOVA) in peroxisomal, mitochondrial, catalase, and SOD2 abundances. Immunofluorescent signals of PEX14 (peroxisomal marker; (**a**–**d**)), ATP5B (mitochondrial marker; (**e**–**h**)), catalase (**i**–**l**), and SOD2 (**m**–**p**) were quantified in distinct neuronal cell types in BoDV1-infected mice of each mouse line (Wt (black), TNFTg (blue), TNFR1ko (red), and TNFR2ko (green) mice). Wt, wild-type; TNFTg, TNF transgenic; TNFR1ko, TNF receptor 1 knockout; TNFR2ko, TNF receptor 2 knockout; BoDV1(+), with BoDV1 infection; CA, cornu ammonis band. Values are means ± SD; * *p* < 0.05, ** *p* < 0.01, *** *p* < 0.001 different from the respective control group.

**Figure 4:**
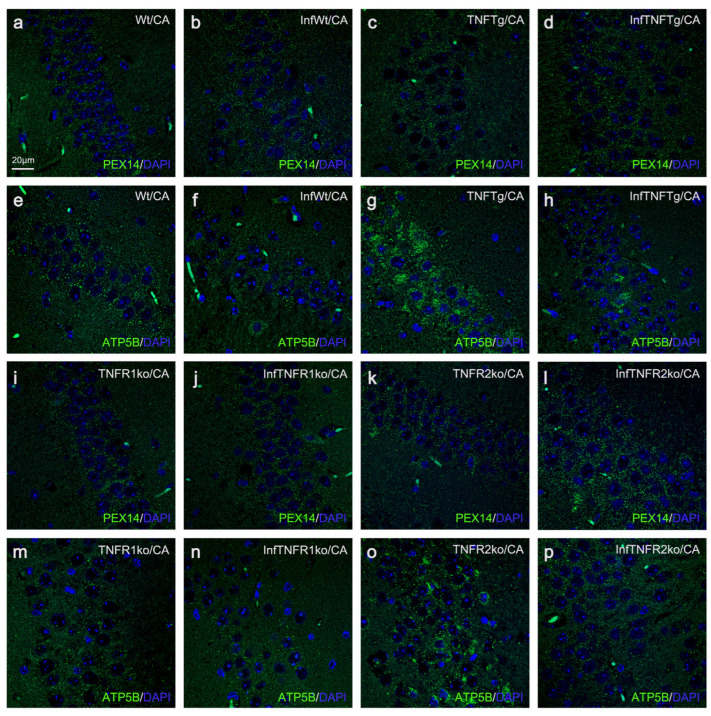
Immunofluorescence photomicrographs of PEX14-positive peroxisomes (green) and ATP5B-positive mitochondria (green) in pyramidal neurons of the CA band of all mouse lines, with and without BoDV1 infection. Using fluorescing antibodies, 2 µm thick brain sections were indirectly immunostained against PEX14 and ATP5B. DAPI (blue) was used as the nuclear stain. Comparison of PEX14-positive peroxisomes between non- and BoDV1-infected mice of the same mouse line: Wt (**a**,**b**), TNFTg (**c**,**d**), TNFR1ko (**i**,**j**), and TNFR2ko (**k**,**l**). Comparison of ATP5B-positive mitochondria between non- and BoDV1-infected mice of the same mouse line: Wt (**e**,**f**), TNFTg (**g**,**h**), TNFR1ko (**m**,**n**), and TNFR2ko (**o**,**p**). Wt, wild-type; TNFTg, TNF transgenic; TNFR1ko, TNF receptor 1 knockout; TNFR2ko, TNF receptor 2 knockout; InfWt, BoDV1-infected Wt; InfTNFTg, BoDV1-infected TNFTg; InfTNFR1ko, BoDV1-infected TNFR1ko; InfTNFR2ko, BoDV1-infected TNFR2ko; CA, cornu ammonis band.

**Figure 5:**
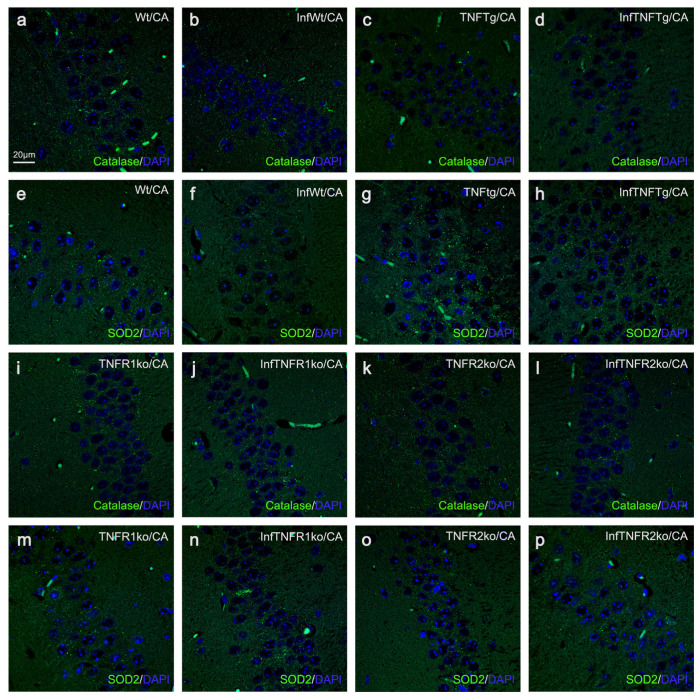
Immunofluorescence photomicrographs of catalase-positive peroxisomes (green) and SOD2-positive mitochondria (green) in pyramidal neurons of the CA band of all mouse lines, with and without BoDV1 infection. Using fluorescing antibodies, 2 µm thick brain sections were indirectly immunostained against catalase and SOD2. DAPI (blue) was used as the nuclear stain. Comparison of catalase-positive peroxisomes between non- and BoDV1-infected mice of the same mouse line: Wt (**a**,**b**), TNFTg (**c**,**d**), TNFR1ko (**i**,**j**), and TNFR2ko (**k**,**l**). Comparison of SOD2-positive mitochondria between non- and BoDV1 infected mice of the same mouse line: Wt (**e**,**f**), TNFTg (**g**,**h**), TNFR1ko (**m**,**n**), and TNFR2ko (**o**,**p**). Wt, wild-type; TNFTg, TNF transgenic; TNFR1ko, TNF receptor 1 knockout; TNFR2ko, TNF receptor 2 knockout; InfWt, BoDV1-infected Wt; InfTNFTg, BoDV1-infected TNFTg; InfTNFR1ko, BoDV1-infected TNFR1ko; InfTNFR2ko, BoDV1-infected TNFR2ko; CA, cornu ammonis band.

Peroxin 14 (PEX14) and adenosine triphosphate synthase F1 subunit β (ATP5B) served as markers for the identification and quantification of peroxisomes and mitochondria, respectively. In non-infected Wt mice, several distinct neuronal cell types showed comparable peroxisomal abundances, ranging from 14/100 µm^2^ in cortical pyramidal neurons (Figure 1a, Figure 2a, Figure S2a and Figure 3c) to 18–21/100 µm^2^ in hippocampal neurons—i.e., granule neurons of dentate gyrus (DG; Figures S1a and S3b) and pyramidal cells of cornu ammonis (CA) band (Figure 1b, Figure 4a and Figure S3a)—and 15–17/100 µm^2^ in cerebellar neurons (i.e., granule and Purkinje neurons; Figure 1c,d, Figure 2c and Figure S3d). About 80% of peroxisomes in hippocampal pyramidal (Figure 1j and Figure 5a) and granule (Figure S1c) neurons contained detectable levels of catalase; values of about 50% were found in cortical pyramidal neurons (Figure 1i, Figure 2i and Figure S2i), and 60% in cerebellar granule neurons (Figure 1k and Figure 2k) as well as in Purkinje neurons (Figure 1l and Figure 2k). Mitochondrial abundance, however, was highest in cerebellar Purkinje neurons (17.5%; Figure 1h, Figure 2g and Figure S3h), followed by cortical pyramidal (9.2%; Figure 1e, Figure 2e, Figures S2e and S3g), hippocampal pyramidal (3.2%; Figure 1f, Figure 4e and Figure S3e), cerebellar granule (2.9%; Figure 1g, Figure 2g and Figure S3h), and hippocampal granule neurons (2.7%; Figures S1b and S3f). Furthermore, 6% and 13% of mitochondria were found to contain SOD2 in hippocampal pyramidal (Figure 1n and Figure 5e) and granule neurons (Figure S1d), respectively; 7% in cortical pyramidal neurons (Figure 1m, Figure 2m and Figure S2m); and 5% and 1% in cerebellar granule (Figure 1o and Figure 2o) and Purkinje neurons (Figure 1p and Figure 2o), respectively.

### 2.2. The Abundances of Peroxisomes, Mitochondria, Catalase, and SOD2 Differ in TNFTg Compared to Wt Mice

In non-infected TNFTg mice, peroxisomal abundances were higher in neurons from brain areas with moderate TNF overexpression compared to respective neurons of non-infected Wt mice: 1.6-fold higher in pyramidal neurons (CA band; Figure 1b and Figure 4a,c) and 1.4-fold in granule neurons (DG; Figure S1a), but not observed in cortical pyramidal neurons (Figure 1a, Figure 2a,b and Figure S2a,c). Further, mitochondrial abundances were higher in all neuronal cell types from TNF-overexpressing brain areas than in the respective neurons of non-infected Wt mice: 5.5-fold in cortical pyramidal neurons (Figure 1e, Figure 2e,f and Figure S2e,g); 9.0-fold in pyramidal neurons (CA band; Figure 1f and Figure 4e,g); and 6.3-fold in granule cells of the DG (Figure S1b). Regarding the antioxidant enzymes, catalase abundance was lower in cortical pyramidal neurons (2.5-fold; Figure 1i, Figure 2i,j and Figure S2i,k), pyramidal neurons of the CA band (3.3-fold; Figure 1j and Figure 5a,c), and granule neurons of the DG (2.6-fold; Figure S1c). SOD2 abundance was higher as well in cortical pyramidal neurons (10.5-fold; Figure 1m, Figure 2m,n and Figure S2m,o), pyramidal neurons of the CA band (11.4-fold; Figure 1n and Figure 5e,g), and granule neurons of the DG (13.7-fold; Figure S1d) from TNF transgenic brain areas of non-infected TNFTg mice compared to same cell types of non-infected Wt mice. Since TNF is expressed only at low levels in the cerebellum of TNFTg mice (used as internal control), no significant differences were found in the abundances of peroxisomes (Figure 1c,d and Figure 2c,d) and mitochondria (Figure 1g,h and Figure 2g,h), as well as the antioxidant enzymes (catalase (Figure 1k,l and Figure 2k,l) and SOD2 (Figure 1o,p and Figure 2o,p)) in cerebellar granule and Purkinje neurons compared to same cell types from Wt mice.

### 2.3. Similar Abundances of Peroxisomes, Mitochondria, Catalase, and SOD2 in Wt and TNFR1ko Mice, but a Higher Organelle Abundance with No Change in Antioxidant Enzymes in TNFR2ko Mice

In non-infected TNFR2ko mice, peroxisomal abundances were higher in neurons of all brain areas compared to respective neurons of non-infected Wt mice: 1.4-fold in cortical pyramidal neurons (Figure 1a and Figure S2a,d); 1.9-fold in pyramidal neurons (CA band; Figure 1b and Figure 4a,k) and 1.8-fold in granule neurons (DG; Figure S1a); and 1.7-fold and 2.1-fold in cerebellar granule (Figure 1c) and Purkinje (Figure 1d) neurons, respectively. At the same time, mitochondrial abundances were higher in cortical pyramidal neurons (2.6-fold; Figure 1e and Figure S2e,h), pyramidal neurons of the CA band (4.7-fold; Figure 1f and Figure 4e,o) and granule neurons of the DG (4.4-fold; Figure S1b), and cerebellar Purkinje neurons (1.8-fold; Figure 1h), except in cerebellar granule neurons (Figure 1g). Despite this overall increase in peroxisomal and mitochondrial abundances in non-infected TNFR2ko mice, catalase abundances (shown in Figure 1i and Figure S2i,l for cortical pyramidal neurons; Figure 1j and Figure 5a,k for pyramidal neurons (CA band); Figure S1c for granule neurons (DG); Figure 1k for granule neurons and Figure 1l for Purkinje neurons of the cerebellar cortex) remained comparable to that of non-infected Wt mice. Likewise, SOD2 abundances in non-infected TNFR2ko mice (shown in Figure 1m and Figure S2m,p for cortical pyramidal neurons; Figure 1n and Figure 5e,o for pyramidal neurons (CA band); Figure S1d for granule neurons (DG); Figure 1o for granule neurons and Figure 1p for Purkinje neurons of the cerebellar cortex) were comparable to that of non-infected Wt mice. Non-infected TNFR1ko mice, however, showed similar peroxisomal abundances (Figure 1a–d, Figure 4a,i and Figure S1a) and mitochondrial (Figure 1e–h, Figure 4e,m and Figure S1b) compared to non-infected Wt mice, and catalase (Figure 1i–l, Figure 5a,i and Figure S1c) and SOD2 (Figure 1m–p, Figure 5e,m and Figure S1d) abundances in both organelles were comparable as well to that of non-infected Wt mice.

### 2.4. Response of Wt Mice to BoDV1 Infection with Regard to Abundances of Peroxisomes, Mitochondria, Catalase, and SOD2

Following BoDV1 infection of Wt mice, peroxisomal abundances increased slightly in all neuronal cell types from the three brain regions (1.5-fold in cortical pyramidal neurons (Figure 1a, Figure 3a and Figure S2a,b); 1.5-fold in pyramidal neurons (CA band; Figure 1b, Figure 3b and Figure 4a,b); 1.3-fold in granule neurons (DG; Figure S1a,e); 1.4-fold in granule neurons (Figure 1c and Figure 3c) and 1.6-fold in Purkinje neurons (Figure 1d and Figure 3d) of the cerebellar cortex; Table 1). Conversely, mitochondrial abundances decreased in all but granule (DG) and cortical pyramidal neurons (2.1-fold in pyramidal neurons (CA band; Figure 1f, Figure 3f and Figure 4e,f); 4.4-fold in granule neurons (Figure 1g and Figure 3g) and 3.8-fold in Purkinje (Figure 1h and Figure 3h) neurons of the cerebellar cortex; Table 1). Next, the abundances of peroxisomal catalase and mitochondrial SOD2 in BoDV1-infected Wt mice were analyzed. A decrease in catalase abundance occurred in all neuronal cell types (3.3-fold in cortical pyramidal neurons (Figure 1i, Figure 3i and Figure S2i,l); 3.3-fold in pyramidal neurons (CA band; Figure 1j, Figure 3j and Figure 5a,b); 3.0-fold in granule cells (DG; Figure S1c,g); 2.9-fold in granule neurons (Figure 1k and Figure 3k) and 4.6-fold in Purkinje (Figure 1l and Figure 3l) neurons of the cerebellar cortex; Table 1). Furthermore, SOD2 abundance decreased in most neuronal cell types—8.1-fold in cortical pyramidal neurons (Figure 1m, Figure 3m and Figure S2m,n); 2.7-fold in pyramidal neurons (CA band; Figure 1n, Figure 3n and Figure 5e,f); 8.0-fold in granule neurons (Figure 1o and Figure 3o) and 3.2-fold in Purkinje neurons (Figure 1p and Figure 3p) of the cerebellar cortex—but not in hippocampal granule neurons of the DG (Figure S1d,h).

### 2.5. Response of TNFtg Mice to BoDV1 Infection with Regard to Abundances of Peroxisomes, Mitochondria, Catalase, and SOD2

After BoDV1 infection of TNFTg mice, we detected no significant changes in peroxisome (Figure 1a,b, Figure 3a,b and Figure S1a,e; Table 1) and catalase (Figure 1i,j, Figure 3i,j and Figure S1c,g; Table 1) abundances in neurons of transgenic areas. This was shown in hippocampal pyramidal neurons, for peroxisomes (Figure 4c,d) and catalase (Figure 5c,d). In the non-transgenic cerebellar cortex, peroxisome abundance remained unchanged (Figure 1c,d and Figure 3c,d), but catalase abundances decreased in granule (3.7-fold) and in Purkinje (3.8-fold) neurons of the cerebellar cortex after BoDV1 infection of TNFTg mice (Figure 1k,l and Figure 3k,l; Table 1). Abundances of mitochondria (Figure 1e–h and Figure 3e–h; Table 1) and SOD2 (Figure 1m–p and Figure 3m–p; Table 1) were 1.8—2.6-fold and 3.0—8.3-fold lower, respectively, in all brain regions except cerebellar granule neurons. Concerning hippocampal pyramidal neurons (CA band), fluorescent micrographs are shown for mitochondria (Figure 4g,h) and SOD2 (Figure 5g,h).

### 2.6. Response of TNFR1- and TNFR2ko Mice to BoDV1 Infection with Regard to Abundances of Peroxisomes, Mitochondria, Catalase and SOD2

Unlike in TNFR1ko mice where peroxisomal and mitochondrial abundances remained unchanged in most neurons after BoDV1 infection (Table 1), mitochondrial abundances decreased 3.4–6.5-fold in all neuronal cell types (Figure 1c–h and Figure 3c–h; Table 1), whereas peroxisomal abundances increased 1.4–1.6-fold further (Figure 1a–d and Figure 3a–d; Table 1), except in cortical pyramidal (Figure 1a, Figure 3a and Figure 4i,j) and cerebellar granule neurons (Figure 1c and Figure 3c; Table 1) in BoDV1-infected TNFR2ko mice. Additionally, catalase (Figure 1j–l and Figure 3j–l; Table 1) and SOD2 (Figure 1m–p and Figure 3m–p; Table 1) abundances decreased markedly, 2.7–10.3-fold and 3.4–9.1-fold, respectively, in BoDV1-infected TNFR2ko mice (shown for hippocampal pyramidal neurons in Figure 5k,l,o,p). Meanwhile, no such changes in catalase abundances (except in cerebellar granule neurons) or in SOD2 abundances were observed in BoDV1-infected TNFR1ko mice (shown for hippocampal pyramidal neurons in Figure 5i,j,m,n; Table 1).

## 3. Discussion

BoDV1 infection of laboratory rodents presents an important opportunity for the extensive study of virus-induced, immune-mediated neuropathological diseases accompanied by viral persistence. Our study investigated the potential changes in peroxisomal, mitochondrial, catalase, and SOD2 abundances in a mouse model with an altered TNF system, before and after BoDV1 infection. Here, we discuss (i) the role(s) of TNF/TNFR1/TNFR2 in relation to the organelle abundances and their main antioxidant enzymes, catalase and SOD2, under physiological conditions; (ii) possible mechanisms underpinning the BoDV1-induced changes in both subcellular compartments in Wt mice; and (iii) and the differences in changes in the abundances of both organelles, catalase and SOD2, after BoDV1 infection of TNFTg, TNFR1ko, and TNFR2ko mice compared to Wt mice, with a particular focus on the role(s) of TNFR1/TNFR2.

### 3.1. Differences in the Abundances of Peroxisomes and Mitochondria as Well as Their Main Antioxidant Enzymes Catalase and SOD2 in the Brains of Wt, TNFTg, TNFR1ko, and TNFR2ko Mice

When comparing organelle abundances in different neuronal cell types of non-infected Wt mice, mitochondrial abundances varied markedly with only minor changes in peroxisomes. The highest abundances of mitochondria were detected in cerebellar Purkinje cells and cortical pyramidal neurons. Perhaps this depicts the variations in energy requirements of different neuronal cell types for metabolism; for instance, projection neurons need sustainable mitochondrial energy support for saltatory conduction and transport of organelles and neurotransmitters along the axon [39].

In non-infected TNFTg compared to Wt mice, chronic overexpression of TNF increased the abundances of peroxisomes in hippocampal pyramidal and cortical neurons—the increase was even higher with regard to the abundances of mitochondria. In regard to the reason for this phenomenon, we assume that TNF signaling generates ROS [40,41] and inhibits peroxisomal α-oxidation [42,43], leading to the accumulation of VLCFAs and activation of the peroxisome proliferator-activated receptor-α (PPAR-α) [44]. In previous studies, increased levels of both ROS [33] and VLCFAs [45] induced peroxisomal proliferation. PPAR-α acts synergistically with PPARγ coactivator-1α (PGC-1α) at the transcriptional level to modulate the abundances and metabolic activities of peroxisomes and mitochondria in neurons [28]. Interestingly, as peroxisomal abundances increased in transgenic brain areas, catalase abundances decreased, suggestive of either depletion or defective import of catalase into peroxisomes as, e.g., found during aging [46]. Similar to our findings, TNF treatment of microglial cells in vitro and the excess of TNF in the brains of multiple sclerosis patients or the respective mouse models reduced catalase levels, resulting in oxidative stress [47].

Mitochondrial abundance increases via mitochondrial fragmentation and biogenesis in both acute [48,49] and chronic [50] exposures of different cell types to TNF. This is due to TNF-induced increases in ROS, endoplasmic reticulum stress, and activation of PGC-1α [28,51]. In addition to these findings, we observed that the number of mitochondria with detectable levels of SOD2 increased in the same proportion in all neurons from TNF-overexpressing brain areas. SOD2 gene expression is predominantly regulated by the transcription factors such as nuclear respiration factor-2, activator protein (AP)-1, and nuclear factor-kappa B (NF-κB) [52]. AP-1 and NF-κB are triggered by the binding of TNF to TNFR1 and TNFR2 [52], which might explain the increase in SOD2 in TNF transgenic brain areas. In another vein, the increase in mitochondrial SOD2 could be a compensatory response to the decrease in peroxisomal catalase based on the well-known crosstalk between both organelles in maintaining intracellular redox homeostasis [28].

Surprisingly, our data on non-infected TNFR1- and TNFR2ko mice revealed that endogenous, physiological (low) levels of TNF very likely increased peroxisomal and mitochondrial abundances via TNFR1 signaling in the absence, but not in the presence of TNFR2, but did not affect the abundances of catalase and SOD2. Here, the role of TNF and TNFRs in the biogenesis of both organelles fits with the inductive functions of the TNF system during cell proliferation and differentiation, especially in progenitor cells [53].

### 3.2. BoDV1 Induced Changes in the Abundances of Peroxisomes, Mitochondria, Catalase, and SOD2 in the Brains of Wt Mice

After BoDV1 infection, peroxisomal abundance increased in most neurons from Wt mice. This increase may reflect the need of peroxisomes for viral membrane assembly. Certain enveloped viruses, such as the RNA influenza virus [54], exploit peroxisomal metabolism to biosynthesize lipids such as plasmalogens for their viral membrane synthesis and replication [26,55]. In addition to the increase in abundance, the peroxisomal matrix enzyme catalase decreased, suggesting a disturbed metabolism causing an adaptive increase in the number of this organelle. Indeed, in horses, BoDV1 infection reduced the level of phosphoethanolamine, pointing to a reduction in peroxisomal lipid metabolism in the hippocampi of asymptomatic animals [56]. Similarly, primary skin fibroblasts from Zellweger patients—which completely lack peroxisomes—demonstrated resistance to infection by enveloped herpesviruses [26,57]. The mechanisms by which enveloped viruses reduce peroxisome numbers after infection of target cells occur via direct interactions between viral proteins and peroxisomal biogenesis proteins (peroxins or PEXs), resulting in a loss of peroxisomal structural integrity, matrix protein content, and ability to function in antiviral immune signaling. For example, the open reading frame (ORF)-1 of SARS-CoV2 was shown to interact with human PEX14 [58]; the capsid proteins of West Nile and Dengue viruses targeted PEX19 [53]; and the NS2A protein of Zika virus bound to PEX3 and PEX19 [59], leading to a decrease in peroxisomes.

The reduction in mitochondrial abundance after BoDV1 infection in Wt mice could be due to either an inhibition of its biogenesis and/or stimulation of mitophagy [27,60,61]. For instance, large enveloped viruses have been shown to hinder mitochondrial biogenesis of the host cells [27,60,61], which might be responsible for their ability to replicate and escape from the innate antiviral immune system [26,27,35,53]. Hepatitis B [62,63] and C viruses [64,65] induced mitochondrial fission and mitophagy to reduce toxic ROS generation from mitochondria, dampen immune response, and persistently infect host cells [27]. The mitochondrion-localized inhibitor of apoptosis of the human cytomegalic virus induced mitochondrial fragmentation and loss, stifling downstream innate immune signaling [66]. Thus, BoDV1-induced decrease in mitochondria could also serve to reduce ROS generation and escape viral immune response, thereby facilitating viral persistence albeit with a small enveloped virus. The decrease in ATP5B protein by BoDV1, however, might reflect not only a reduction in mitochondrial abundance but also an inhibition of the respiratory chain activity (of complex V), as observed during infection with SARS-CoV-2 [67] and hepatitis B virus protein [68]. Both changes were thought to be caused by mitochondria-derived ROS, which interestingly requires peroxisomal metabolism as it does not occur in peroxisome (PEX5)-deficient mice [69].

After entry of a virus into a host cell, viral nucleic acids and proteins are recognized by pattern recognition receptors (PRRs) in the endosome or cytosol [27,70,71]. Binding of viral RNAs to PRRs such as Toll-like receptors (TLRs; for ssRNAs like that of BoDV1 by TLR7/8) and RLRs leads to the activation NF-κB and IFN regulatory factors [70,71]. This causes the release of IL-6, IFNβ [70], and TNF [71]; the latter activates pro-inflammatory pathways of NF-κB, and in this way, a vicious cycle and cytokine storm arise, recruiting immune cells (mainly T-cells in the case BoDV1 infection, although BoDV1 suppress RIG activation) to the site of infection [8,71]. Peroxisomal and mitochondrial MAVSs serve as supramolecular organizing centers for interaction and transmission of antiviral signals from RLRs, leading to the synthesis and release of IFN types I and III and/or pro-inflammatory cytokines [72,73]. In fact, the X protein of BoDV1 has been shown to interact with MAVSs on mitochondria, curtailing both the antiviral response engineered by this organelle and neuronal apoptosis, which is believed to help sustain perpetual infection of brain cells [36,74]. In another study, persistent BoDV1 infection downregulated microRNA (miR-155) levels via its phosphoprotein, hindering the release of type I IFNs and consequently the innate antiviral immune signaling [75]. Of note, different strains of BoDV have been shown to produce differing pathophysiological effects in vitro or in vivo and in different species of animals [76]. The precise mechanisms on how BoDV1 infection caused mitochondrial loss in neurons in our study, and whether this enhances viral persistence in the CNS, require further investigations.

### 3.3. Differences in Responses after BoDV1 Infection of TNFTg, TNFR1ko, and TNFR2ko Mice in Comparison to Wt Mice with Regard to the Abundances of Peroxisomes and Mitochondria, Catalase, and SOD2

Upon viral infection, previously elevated levels of TNF and subsequent neuroinflammation can either facilitate neurodegeneration or exert neuroprotection [24]. Thus, we used TNFTg mice in the present study to investigate whether prolonged in vivo exposure of several distinct neuronal cell types to TNF influences the peroxisomal and mitochondrial compartments after BoDV1 infection. Furthermore, TNFR1ko and TNFR2ko mouse lines were used to address the same question concerning the role of TNFR1 and TNFR2 signaling.

After BoDV1 infection of TNFTg mice, peroxisomal and catalase abundances did not change in neuronal cells from transgenic brain areas, suggesting that mild chronic TNF pre-sensitization of neurons in vivo counteracted changes in peroxisomal and catalase abundance after BoDV1 infection. Unexpectedly, in the cerebellar cortex of TNFTg mice with very low TNF levels like in Wt mice, catalase abundances decreased in both Purkinje and granule neurons whilst SOD2 abundance increased in Purkinje neurons but remained unchanged in granule neurons. Of note, there seems to be a reactionary, counteractive response by the mitochondrial compartment to the decreased antioxidant capacity of peroxisomes in the non-transgenic cerebellar cortex after BoDV1 infection, which enriches the increasing evidence on the significant redox-based relationship between peroxisomes and mitochondria [77,78]. The abundance of peroxisomes increased whereas that of catalase decreased following BoDV1 infection in Wt and TNFR2ko mice; these changes were not observed in TNFR1ko mice. This incriminates TNFR1 signaling as a major role-player in the observed BoDV1-driven changes in the peroxisomal and mitochondrial compartments.

In BoDV1-infected TNFTg and TNFR1ko mice, mitochondrial and SOD2 abundances decreased, although both were still at higher levels than found in BoDV1-infected Wt mice. Our findings buttress the generally accepted, overall protective and pro-survival roles of TNFR2 [8,22,23]. In other studies, transmembrane TNF/TNFR2 (in the absence of TNFR1) curtailed the ischemic destruction of retinal tissue [79] and autoimmune encephalomyelitis [80]. Additionally, Wt and TNFR1ko mice pre-sensitized with TNF or agonistic TNFR2-specific antibodies were tolerant to glutamate excitotoxicity, whereas neurons from TNFR2ko died after glutamate and/or TNF treatments [24]. Similarly, after infection with *Listeria monocytogenes*, TNFR2 signaling enhanced the survival and clonal expansion of CD4+ and CD8+ T cells, thereby promoting pathogen elimination and neuroprotection [81].

As part of attempts by neurons to eliminate microbes, ROS are generated, followed by an increase in their antioxidant enzymes. Depending on the degree or duration of inflammation, neurons might lose their capacities to detoxify rising levels of ROS; then, oxidative stress sets in with deleterious damage to subcellular organelles and their functions, even leading to organelle and cell death [22,27,30]. The decrease in catalase and SOD2 in different brain regions after BoDV1 infection, particularly in Wt and TNFR2ko mice, very likely destabilizes intracellular redox homeostasis, leading to oxidative stress and mitochondrial damage and loss. This, together with microglial activation and unusual astrogliosis [8,15], might contribute to the development of seizures and non-purulent meningoencephalitis in BoDV1-infected neonatal mice.

Overall, our results show that TNFR1 signaling mediated the BoDV1-induced changes in the abundances of peroxisomes and mitochondria, as well as their main antioxidant enzymes. This provides additional insight into the possible mechanisms by which BoDV1 maintains persistent infection in the CNS, which might lead to the development of new therapeutic strategies for BD in humans and animals.

## 4. Materials and Methods

### 4.1. Materials

Ethanol was obtained from SAV LP Co., Ltd. (Flintsbach am Inn, Germany); non-buffered formalin and xylene from Carl Roth Co., Ltd. (Karlsruhe, Germany); Dulbecco’s Modified Eagle’s Medium (DMEM, 1 g/L D-glucose) and 2% heat-inactivated bovine serum from Thermo Fisher Scientific (Dreieich, Germany); and DAPI (4′, 6-diamino-2-phenylindole), bovine serum albumin (BSA), and Tween 20 from Sigma-Aldrich (Deisenhofen, Germany). Details of the primary antibodies used in this study are provided in Table 2. Goat anti-rabbit IgG Alexa Fluor 488 (1:300; Invitrogen by Thermo Fisher Scientific, Dreieich, Germany) was used as the secondary antibody for indirect immunofluorescence staining.

### 4.2. Methods

#### 4.2.1. Mouse Lines and Genotyping

Wild-type mice (C57BL/6JOlaHsd) were obtained from Harlan Laboratories Co., Ltd., Indianapolis, IN, USA; while TNFTg mice (C57Bl/6-Tg(Grin2b-Tnf)41.3MK), TNFR1ko mice (B6.129-Tnfrsf1a tm1Blt/J), and TNFR2ko mice (B6.129S2-Tnfrsf1btm1Mwm/J) were kindly provided by Prof. Dr. Ulrich L.M. Eisel, Department of Molecular Neurobiology, University of Groningen, Netherlands. For each mouse line, male and female mice were used with no special attention to equal sex distribution. In TNFTg mice, the murine TNF gene is under the control of the NR2B subunit of the N-methyl-D-aspartate (NMDA) glutamate receptor promoter, resulting in selective TNF overexpression in the hippocampus, neocortex, thalamus, and striatum [8,24,38]. In TNFR1ko mice, exons 2, 3, and part of exon 4 of the TNFR1 gene were replaced by a neomycin resistance cassette [82] and, in TNFR2ko mice, exon 2 (the signal binding region of TNFR2) was deleted and the neomycin resistance cassette was inserted [83]. For genotyping of the alleles, DNA from mouse tails was isolated with Puregene^®^ Core Kit A (Qiagen GmbH, Hilden, Germany), and a multiplex end-point quantitative polymerase chain reaction was performed, as described previously [8,15]. All animals were bred and housed at the central animal laboratory of Justus Liebig University Giessen in accordance with animal welfare regulations, and all animal experiments were performed with Hessen state approval (Regional Council, Giessen, file number V54-19c 2015(1) GI18/4 No. 12/2012). Mice, including those experimentally infected with BoDV1, were kept at the animal facility of Biosafety Laboratories 3 of Philipps University Marburg.

#### 4.2.2. Preparation of BoDV1 Suspension and Experimental Infection of Mice

The original BoDV-1 stock (H24), prepared from 4-week-old Lewis rats and passaged 4 times in Balb/c mouse brains [13], was injected into the brains of neonatal heterozygous TNFTg mice. Brain extracts were diluted to reach a final virus titer of 5 × 10^5^ ID_50_/mL [15]. Each mouse line was split into two groups: (i) a non-infected group, where mice were not exposed to BoDV1, to serve as controls (8 Wt, 5 TNFTg, 5TNFR1ko, and 8 TNFR2ko mice); (ii) and an infected group (8 Wt, 5 TNFTg, 5 TNFR1ko, and 7 TNFR2ko mice), where mice were infected with BoDV1 via intracerebral injection on day 0. The BoDV1 suspension as described above was diluted in DMEM (1:100) at 37 °C. At normal body temperature, 1 μL of the diluted viral suspension vehicle was injected into the left front hemisphere of the brain using a 26-gauge Hamilton^®^ syringe (Hamilton Company, Bonaduz, Switzerland). The injection site was identified using a line that connects the lateral corners of the eyes, paramedian to the left; the penetration depth was regulated by a stopper on the cannula. Viral nucleoprotein (BoDV1-N) was detected in the entire brain in the majority of neuronal cells between 35 and 42 dpi [8]; hence, all mice used in this study were 42 days old.

#### 4.2.3. Brain Tissue Perfusion and Preparation

At 42 dpi, all mice, with or without BoDV1 infection, were humanely euthanized as described elsewhere [15]. For each mouse, the scalp and calvaria were opened with sterile scissors, and the brain was removed with a sterile spoon and divided sagittally into two halves. Both hemispheres were fixed in 10% unbuffered formalin for approximately 24 h. On the next day, brain hemispheres were divided into three transverse planes (Figure S4a) containing the hippocampal, cerebral, and cerebellar cortices—as described previously [8,84]—and embedded in paraffin.

#### 4.2.4. IF Staining of Formalin-Fixed, Paraffin-Embedded (FFPE) Mouse Brain Tissue

Using a microtome, 2 µm thick sections of the FFPE brain tissue were cut onto Superfrost^®^ plus microscope glass slides (R. Langenbrinck Co., Ltd., Emmendingen, Germany), deparaffinized in xylene (3 × 5 min), and rehydrated by decreasing concentrations of ethanol (99%, 99%, 96%, 80%, 70%, and 50%) and, finally, in distilled water (2 × 3 min). Antigen retrieval was performed by incubating the slides in 0.01% trypsin for 10 min at 37 °C followed by heating in citrate buffer (pH = 6.0) for 3 × 5 min in a standard microwave at 800 W with washing steps in between. Nonspecific binding sites were blocked with 4% BSA and 0.05% Tween 20 in phosphate-buffered saline (PBS) for 2 h. Sections were washed in PBS for 3 × 5 min prior to incubation with primary antibodies overnight. On the following day, brain sections were washed with PBS for 3 × 5 min prior to incubation with secondary antibodies for 2 h at room temperature. Afterwards, sections were washed again with PBS for 3 × 5 min. Nuclei were stained with DAPI.

#### 4.2.5. Fluorescence Microscopic Morphometry of Peroxisomes and Mitochondria and Their Antioxidant Enzymes

PEX14, a peroxisomal membrane protein, is an optimal marker for the identification and quantification of peroxisomes in different cell types because its expression is constant and independent of cellular metabolism [85,86]. ATP5B is an essential component of the mitochondrial membrane complex and a regulator of mitochondrial fission/fusion [87]; thus, ATP5B was used for the quantification of the mitochondrial network. In addition, catalase and SOD2 were used as markers to investigate the antioxidant capacity of peroxisomes and mitochondria, respectively.

Using a Leica confocal laser-scanning microscope (type SPC2; 63× objective), photomicrographs were taken from the (i) dentate gyrus and CA band regions (mainly in CA2 and CA3, because BoDV1 spread to CA1 was rare) of the hippocampus; (ii) cerebral motor cortex (laminae III (external pyramidal neurons) and V (internal pyramidal neurons)); and (iii) cerebellar cortex (Figure S4b,c). In the hippocampus and cortex, we further distinguished between granule and pyramidal neurons; and, in the cerebellum, Purkinje neurons were additionally evaluated, as they are suitable representations of the major cell types present in the three different brain regions for quantitative analyses. Fiji Image J software (http://imagej.nih.gov/ij) was used to quantify the signals of PEX14, ATP5B, catalase, and SOD2 in all photomicrographs. Regarding peroxisomes, the abundance of this organelle denotes the number area density of PEX14-positive peroxisomes. This was calculated by counting the number of PEX14 signals (n), measuring the respective cytosolic area (A), and computing the abundance as n/A100 µm^2^ (Figure S4d–h,n)—the same was performed for catalase abundance, where catalase-positive peroxisomes represented only those with a high antioxidant capacity. Mitochondrial and SOD2 signals appeared as a diffused network; hence, we measured the area of the fluorescence signals of ATP5B (representing all mitochondria) and SOD2-positive mitochondria (only those ones with a high antioxidant capacity) and the cytosolic area. Abundances of mitochondria and SOD2, which denote the percentage (%) area of mitochondria and SOD2, respectively, were calculated as shown in Figure S4i–m,o.

#### 4.2.6. Statistical Data Analysis

For each neuronal cell type in a defined brain region, we evaluated differences between Wt, TNFTg, TNFR1ko, and TNFR2ko mice—with (Figure 3 and Figure S1e–h) or without (Figure 1 and Figure S1a–d) BoDV1 infection—using Brown Forsythe and Welch’s one-way analysis of variance (ANOVA), followed by post hoc Games–Howell multiple comparisons (α = 0.05). If differences between non-infected and BoDV1-infected mice of the same mouse line and neuronal cell type in a defined area were to be assessed, an unpaired, non-parametric Welch’s *t*-test analysis (α = 0.05) was performed (Table 1). Statistical analysis was carried out using GraphPad Prism software (v. 8.0.2; 2019). All data are given as means +/− standard deviation of 8 Wt, 5 TNFTg, 5 TNFR1ko, and 8 TNFR2ko mice for the non-infected mouse groups, and 8 Wt, 5 TNFTg, 5 TNFR1ko, and 7 TNFR2ko mice for those infected with BoDV1. Where there were no significant differences, values of *p* were ≥0.05. All marked changes described in this study are of statistical significance where *p* < 0.05.

## 5. Conclusions

BD, a zoonosis, is increasingly becoming a disease of public health concern. Clinical and virological evidence postulate that viruses that are able to establish persistent CNS infection contribute meaningfully to human mental illnesses of unknown etiologies. Currently, there are neither approved vaccines against BD nor drugs for treatment. Hence, it is unequivocally relevant to explore ways to curb not only the spread of BoDV1 but also the resultant mostly fatal disease.

Our findings show that the TNF system may be involved in the biogenesis of both organelles at endogenous, physiological levels of TNF in neurons. Moreover, BoDV1 infection and exposure of brain cells to excess TNF in vivo altered the antioxidant capacity and abundance of peroxisomes and mitochondria in mice. We suppose that the weakening of the antioxidant capacity of both organelles after BoDV1 infection may cause a shift towards a pro-oxidant redox state, injure cellular components, and induce neuroinflammation. On the other hand, the loss of mitochondria/mitophagy (to avoid neuronal apoptosis and dampen downstream innate antiviral signaling) and the increase in peroxisomes (to enhance viral membrane assembly, replication, and spread) are most likely part of the mechanisms used by BoDV1 for sustaining viral persistence in the brain. BoDV1 infection had little or no effect on mitochondria and peroxisomes, as well as their respective antioxidant enzymes in TNFR1Ko mice, highlighting the altogether protective and pro-survival roles of TNFR2 signaling; hence, the selective blockage of TNFR1 might offer a promising strategy for alleviating BoDV1-mediated CNS pathologies in BD patients. Further investigations involving other dead-end (e.g., humans, horses, and sheep) and reservoir hosts (e.g., shrews) might reveal the importance of these BoDV1-driven changes in the peroxisomal and mitochondrial compartments in the pathomechanisms of BoDV1 infections.

## Figures and Tables

**Table 1:** Welch’s *t*-test comparison of peroxisomal, mitochondrial, catalase, and SOD2 abundances between non- and BoDV1-infected mice of the same mouse line and from the same brain region.

		Mouse Lines		
	Wt vs. InfWt	TNFTg vs. InfTNFTg	TNFR1ko vs. InfTNFR1ko	TNFR2ko vs. InfTNFR2ko
**PEX14**				
GrDG	*	n.s.	n.s.	n.s.
PyCA	*	n.s.	n.s.	*
PyCx	*	n.s.	n.s.	*
GrCb	**	n.s.	n.s.	n.s.
PuCb	*	n.s.	n.s.	*
**ATP5B**				
GrDG	n.s.	**	n.s.	****
PyCA	*	*	n.s.	**
PyCx	n.s.	***	*	***
GrCb	*	n.s.	n.s.	*
PuCb	**	*	n.s.	***
**Catalase**				
GrDG	****	n.s.	n.s.	**
PyCA	****	n.s.	n.s.	****
PyCx	****	n.s.	n.s.	***
GrCb	**	*	**	****
PuCb	***	**	n.s.	****
**SOD2**				
GrDG	n.s.	***	n.s.	**
PyCA	*	*	n.s.	*
PyCx	*	**	n.s.	**
GrCb	*	n.s.	n.s.	*
PuCb	*	*	n.s.	***

PEX14 and ATP5B served as markers for identifying peroxisomes and mitochondria, respectively, within several distinct neuronal cell types. GrDG, granule neurons of dentate gyrus; PyCA, pyramidal neurons of cornu ammonis band; PyCx, pyramidal neurons of cerebral cortex; GrCb, granule neurons of cerebellar cortex; PuCb, Purkinje neurons of cerebellar cortex. Wt, wild type; TNFTg, TNF transgenic; TNFR1ko, TNF receptor 1 knockout; TNFR2ko, TNF receptor 2 knockout; InfWt, BoDV1-infected Wt; InfTNFTg, BoDV1-infected TNFTg; InfTNFR1ko, BoDV1-infected TNFR1ko; InfTNFR2ko, BoDV1-infected TNFR2ko. Significant differences: * *p* < 0.05, ** *p* < 0.01, *** *p* < 0.001, **** *p* < 0.0001. *p* ≥ 0.05 (non-significant (n.s.)).

**Table 2:** Primary antibodies used for the indirect immunofluorescence staining.

Target Antigen	Host	Source; Catalogue Number	Dilution
PEX14	Rb	Kindly donated by Denis I. Crane, Griffith University, Brisbane, Australia	1:10,000
Catalase	Rb	Kindly donated by Denis I. Crane, Griffith University, Brisbane, Australia	1:5000
ATP5B	Rb	Sigma-Aldrich, Deisenhofen, Germany; APA001520	1:2000
SOD2	Rb	Abcam, Cambridge, United Kingdom; ab13533	1:1000

Rb, rabbit.

## Data Availability

All data generated from this study are either included in the main text of this article or provided as supplementary information.

## Supplementary Materials

The following supporting information can be downloaded at: https://www.mdpi.com/article/10.3390/ijms25031849/s1.

## Abbreviations

AP-1	activator protein-1
ATP5B	adenosine triphosphate synthase F1 subunit beta
BD	Borna disease
BoDV1	Borna disease virus 1
InfTNFR1ko	BoDV1-infected TNFR1ko
InfTNFR2ko	BoDV1-infected TNFR2ko
InfTNFTg	BoDV1-infected TNFTg
InfWt	BoDV1-infected Wt
BSA	bovine serum albumin
CA	cornu ammonis
Cb	cerebellar cortex
CNS	central nervous system
Cx	cerebral cortex
DAPI	4′,6-diamidino-2-phenylindole
DG	dentate gyrus
DMEM	Dulbecco’s modified Eagle’s medium
dpi	days post-infection
H_2_O_2_	hydrogen peroxide
IFN	interferon
IL	interleukin
IRFs	IFN regulatory factors
MAVS	mitochondrial antiviral signaling protein
NF-κB	nuclear factor kappa B
NR2B	N-methyl-D-aspartate 2b receptor subtype
PBS	phosphate-buffered saline
PEX	peroxin
PGC-1α	peroxisome proliferator-activated receptor gamma coactivator 1 alpha
PPAR	peroxisome proliferator-activated receptor
RLRs	retinoic-acid-inducible gene I (RIG-I)-like receptors
ROS	reactive oxygen species
SOD	superoxide dismutase
TNFR	tumor necrosis factor-α receptor
TNFR1ko	TNF receptor 1 knockout
TNFR2ko	TNF receptor 2 knockout
TNFTg	TNF transgenic
TNF	tumor necrosis factor alpha
VLCFA	very-long-chain fatty acid
Wt	wild type
