# Supplementary material for: Tumor Necrosis Factor-α Receptor 1 Mediates Borna Disease Virus 1-Induced Changes in Peroxisomal and Mitochondrial Dynamics in Neurons
Source: Int J Mol Sci. 2024 Feb 3;25(3):1849. doi: 10.3390/ijms25031849 (PMC10855776; doi:10.3390/ijms25031849)
Supplement: Supplementary file 1 [file ijms-25-01849-s001.zip › ijms-2814692-supplementary.pdf]

List of Supplementary Figures

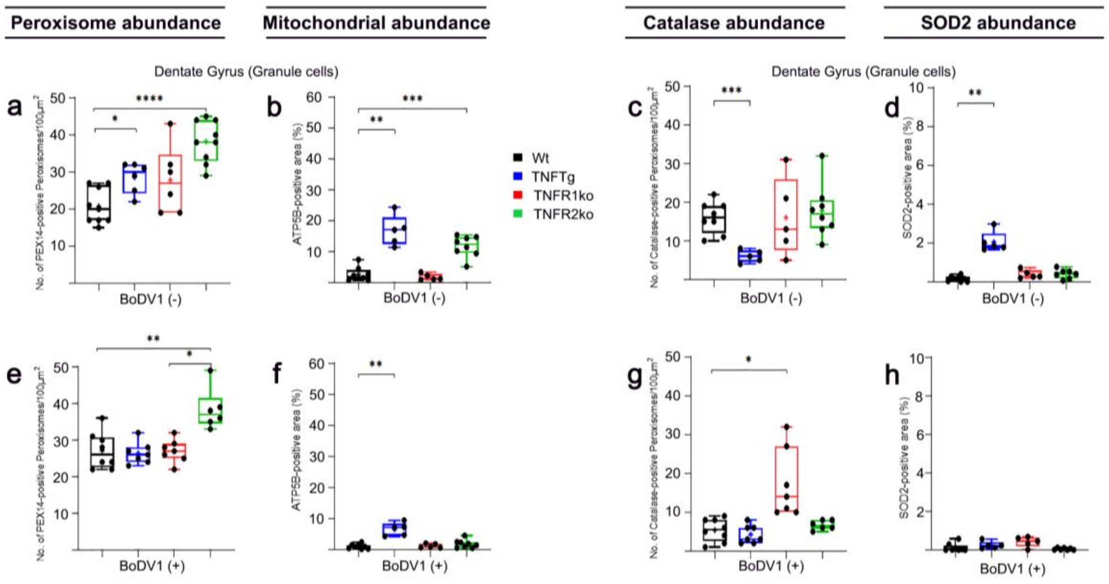

Figure S1. Differential changes (one-way ANOVA) of PEX14 (a, e)- and catalase (c, g)-positive peroxisomes, ATP5B (b, f)- and SOD2 (d, h)-positive mitochondria in granule neurons of the dentate gyrus of different mouse lines, with and without BoDV1 infection (Wt (black), TNFTg (blue), TNFR1ko (red) and TNFR2ko (green) mice). Wt, wild-type; TNFTg, TNF transgenic; TNFR1ko, TNF receptor 1 knockout; TNFR2ko, TNF receptor 2 knockout; BoDV1(-), without BoDV1 infection; BoDV1(+), with BoDV1 infection. Values are means ± SD; * $p < 0.05$, ** $p < 0.01$, *** $p < 0.001$, **** $p < 0.0001$ different from the respective control group.

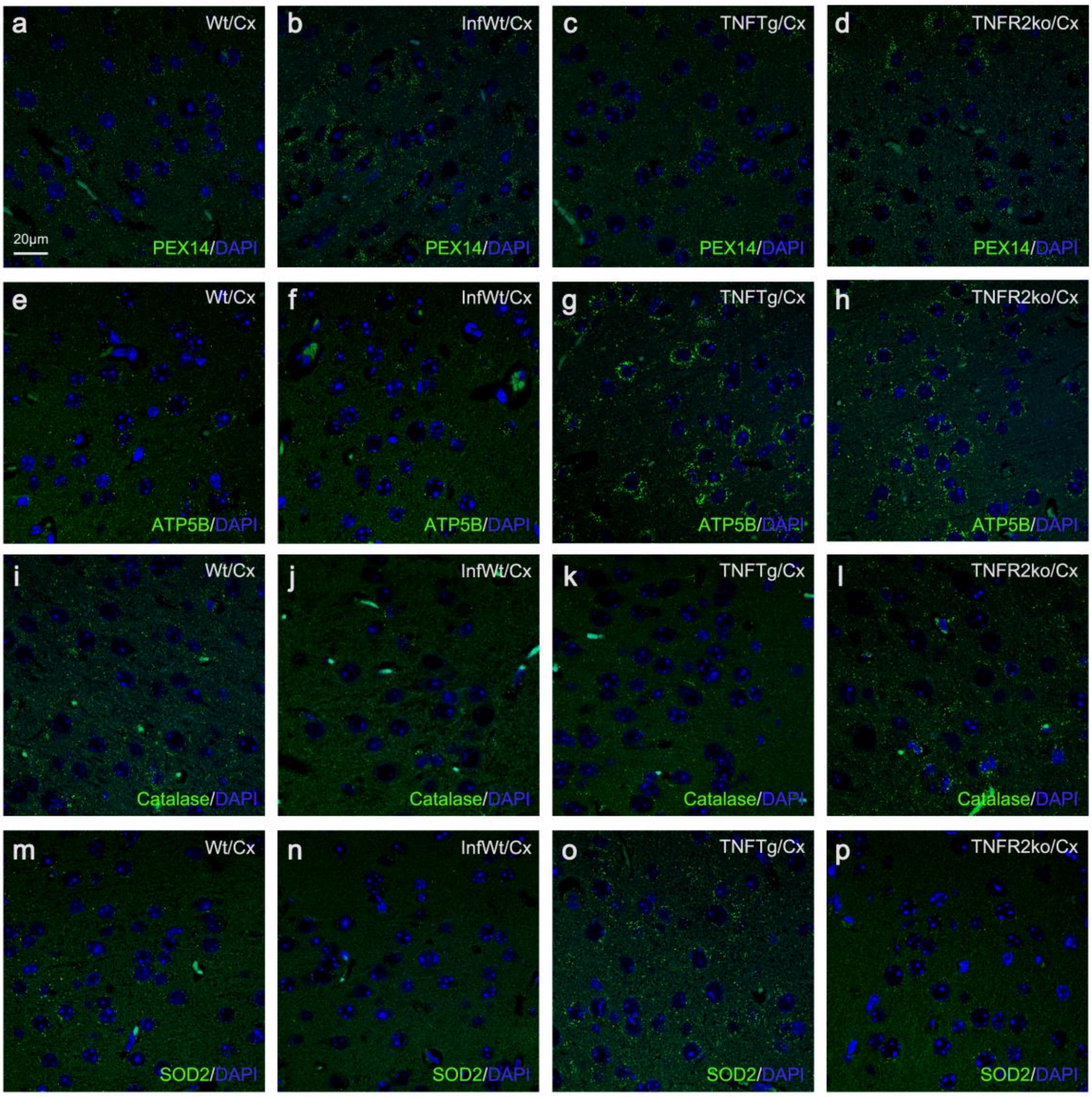

Figure S2. Immunofluorescence photomicrographs of PEX14 (a–d)- and catalase (i–l)-positive peroxisomes as well as ATP5B (e–h)- and SOD2 (m–p)-positive mitochondria in pyramidal neurons of the cerebral cortex of non-infected and BoDV1-infected mice. Brain sections (2µm) were stained using primary antibodies against PEX14 (peroxisomal marker), ATP5B (mitochondrial marker), catalase, and SOD2 and secondary antibodies coupled with Alexa Fluor® 488 (green) together with the nuclear dye DAPI (blue). Wt, wild-type; TNFTg, TNF transgenic; TNFR2ko, TNF receptor 2 knockout; InfWt, BoDV1-infected Wt; Cx, cerebral cortex.

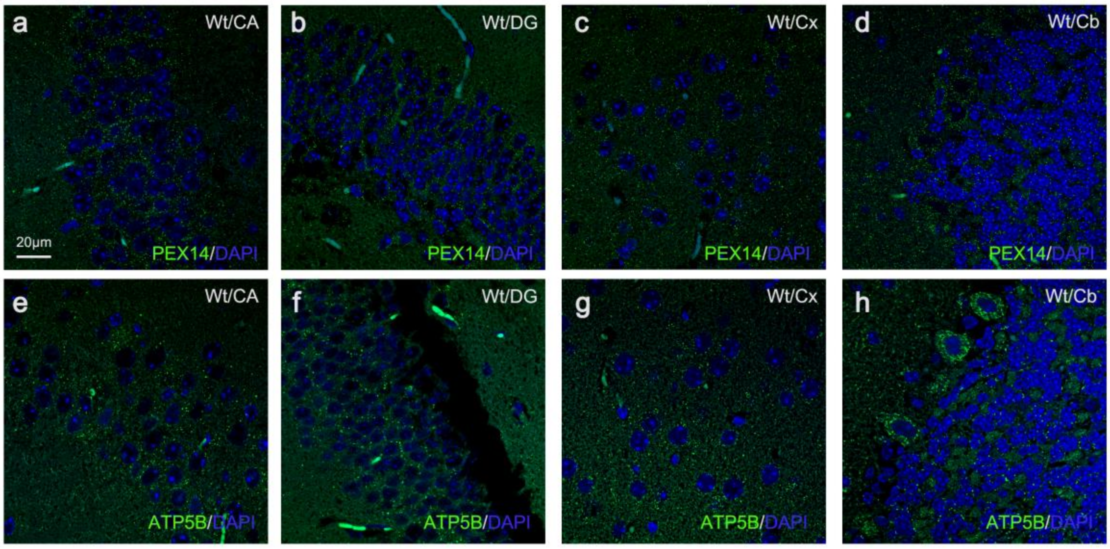

Figure S3. Immunofluorescence photomicrographs of PEX14-positive peroxisomes (a–d) and ATP5B-positive mitochondria (e–h) in several distinct neuronal cell types of the hippocampus (DG and CA band), cerebral and cerebellar cortices of non-infected Wt mice. Using fluorescing antibodies, 2μm-thick brain sections were indirectly immunostained against PEX14 (peroxisomal marker) and ATP5B (mitochondrial marker). DAPI (blue) was used as the nuclear stain for all neuronal cell types. Wt, wild-type; CA, cornu ammonis band; DG, dentate gyrus; Cx, cerebral cortex; Cb, cerebellar cortex.

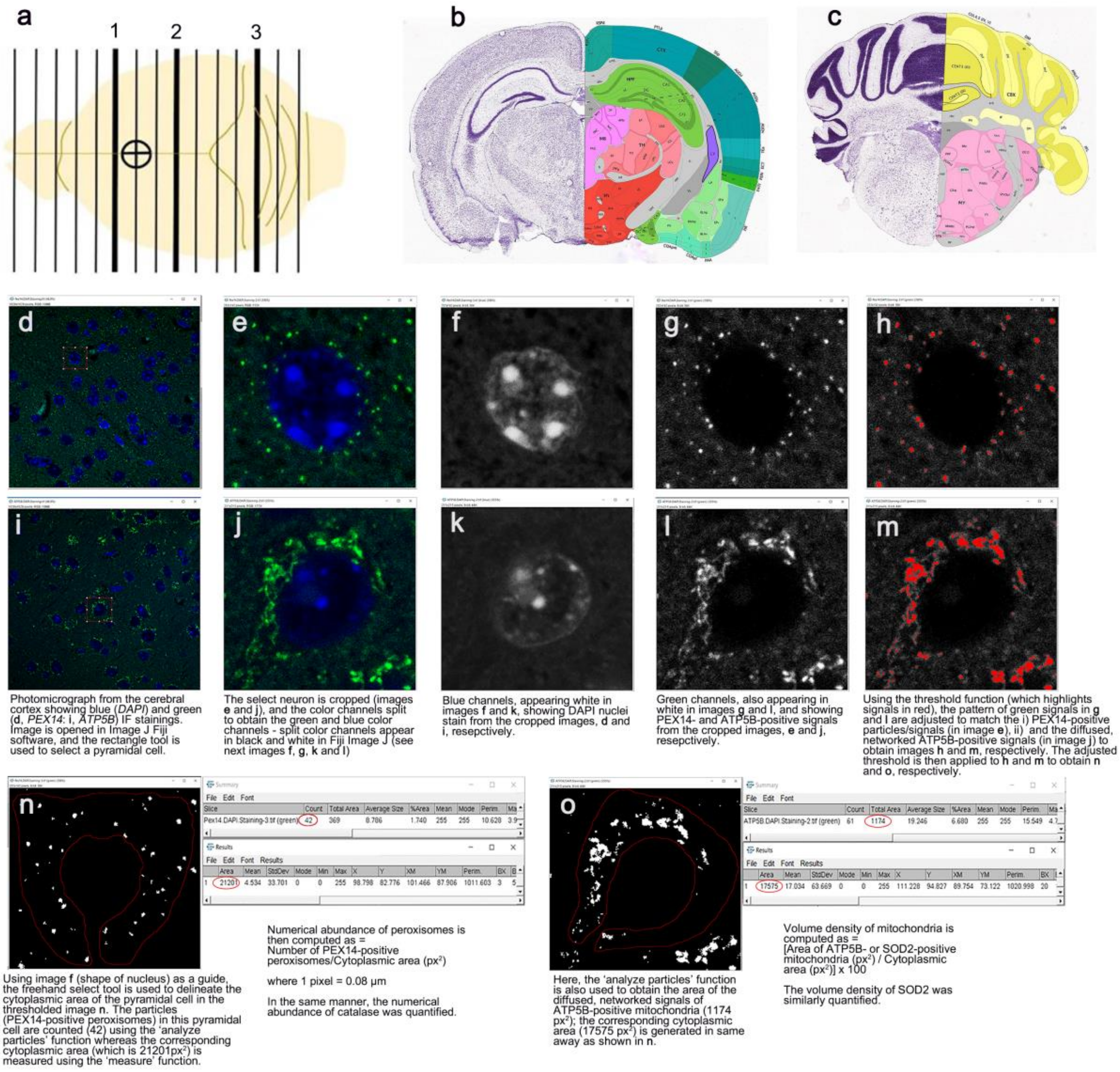

Figure S4. Schematic diagram showing dorsal view of mouse brain divided transversely (shown by thicker black lines) at levels: 1 (B; Bregma 1.32 mm; Interaural 5.12mm), 2 (C; Bregma -2.12mm; Interaural 1.68 mm) and 3 (D; Bregma -5.88; Interaural -2.08) to obtain the three brain areas (a; modified from atlas by T. Capra). Bregma (the cross inside a circle) is the point where the coronary suture and the sagittal suture intersect. Distance between bars is 1 mm. In the hippocampal formation (HPF), granule neurons were analyzed from the dentate gyrus (DG) and pyramidal neurons from the cornu ammonis (CA) bands 3 and 2; pyramidal neurons from laminae III and V were analyzed from the cerebral mortor cortex (b; Allen brain atlas). Granule and Purkinje neurons from the cerebellar cortex (CBX) were also analyzed (c; Allen brain atlas). Quantitative analyses of the abundances of PEX14-positive peroxisomes (d–h, n) and ATP5B-positive mitochondria (i–m, o).
